# Effect of Real-Time Environment on Mechanical Properties of Preformed Stainless Steel Archwires: An In Vivo Study

**DOI:** 10.1155/2023/5882121

**Published:** 2023-04-11

**Authors:** Divya Pai, Arun S. Urala, Kishore Ginjupalli, Kalyana Chakravarthy Pentapati, Gaurav Agrawal

**Affiliations:** ^1^Department of Orthodontics and Dentofacial Orthopaedics, Manipal College of Dental Sciences, Manipal Academy of Higher Education, Manipal 576104, India; ^2^Department of Dental Materials, Manipal College of Dental Sciences, Manipal Academy of Higher Education, Manipal 576104, India; ^3^Department of Public Health Dentistry, Manipal College of Dental Sciences, Manipal Academy of Higher Education, Manipal 576104, India; ^4^Department of Orthodontics and Dentofacial Orthopaedics, Rungta College of Dental Sciences and Research, Bhilai 490024, India

## Abstract

**Introduction:**

Clinicians should be aware of any effect the oral environment may have on archwires. Laboratory models fail to closely imitate intraoral conditions. The aim was to evaluate the change in mechanical properties of preformed stainless steel archwires after 15 weeks of exposure to the oral environment.

**Methods:**

Three commercially manufactured 0.019 × 0.025″ stainless steel archwires were evaluated. Young's modulus, yield strength, spring factor, and hardness were studied. The unexposed distal end cuts (control samples) and archwires were tested after 15 weeks of intraoral exposure (test samples). Tension tests, Vickers microhardness tests, and nanoindentation tests were carried out.

**Results:**

Normality was tested using the Shapiro–Wilk test. Statistical analyses included the paired *t*-test for intragroup comparisons and Kruskal–Wallis ANOVA with the post hoc Dunn test for comparison of mean percentage reduction in values. At T15, Young's modulus showed a statistically significant decrease. Changes in yield strength and spring factor were not significant for groups other than American Orthodontics wires. The reduction in hardness was significant in 3M Unitek. Vickers, tension, and nanoindentation tests demonstrated an expansive range between hardness and Young's modulus so determined.

**Conclusion:**

3M Unitek archwires showed the highest difference in Young's modulus. Yield strength values increased in Ortho Organizers archwires. Spring factor decreased only in 3M Unitek archwires. Hardness values obtained from various tests did not produce identical results.

## 1. Introduction

The application of precise orthodontic force systems is essential for good control over tooth movement. When selecting a suitable archwire, an orthodontist needs to consider an array of mechanical properties such as spring back, yield strength, and elastic modulus [1]. Austenitic stainless steel is most commonly favoured due to its corrosion resistance, good formability, high stiffness, resilience, and moderate cost [2].

However, even after extensive use, the mechanical properties of these wires remain uncertain [3]. The oral environment could be responsible for this uncertainty as the current properties of wires are deduced from testing in vitro. The clinician should be aware of any effect that the oral environment could have on the properties of orthodontic archwires as they are vulnerable to intergranular attack and stress corrosion due to the presence of carbon and molybdenum [4]. The oral cavity is known for the presence of complex oral flora and plaque [5]. The currently available in vitro methods fail to simulate this multifaceted intraoral environment, leading to a significant shortage of information concerning the intraoral aging of orthodontic materials.

Microhardness testing methods were popular in the past for studying wire properties. An emerging shift is where nanoindentation is used to study the mechanical properties of commercially available archwires [6]. However, evaluation of the mechanical properties of stainless steel wires produced by different manufacturers needs to be conducted as wire fabrication by cold working [7] manipulates properties of these wires. Hence, characterizing these properties will help the clinician in predicting accurate results. The study aimed to evaluate the mechanical properties of three commercially available preformed stainless steel wires after 15 weeks of exposure to the oral environment.

## 2. Materials and Methods

### 2.1. Ethics Approval

The study protocol was approved by the Institutional Ethics Committee at Kasturba Hospital, Manipal (IEC: 726/2014). All procedures were performed in compliance with relevant laws and institutional guidelines. Informed consent was obtained from all the participants.

### 2.2. Sample Size Estimation

We have conducted a pilot study to evaluate the feasibility of the experiment among five samples in each group. The mean values of MOE (Table 1) obtained were 169.1, 163.3, and 183.2 among the three groups of wires with a SD of 14.1. This yielded an effect size of 0.6. The sample size was estimated using G∗Power software (version 3.1.9.4). A total of 30 subjects were required (*n* = 10 in each group) with a power of 80% and 95% confidence intervals.

### 2.3. Sample Preparation

The samples consisted of 0.019″ × 0.025″ stainless steel orthodontic archwires obtained from three different manufacturers American Orthodontics (Sheboygan, Wisconsin, USA), 3M Unitek (Monrovia, California, USA), and Ortho Organizers (San Marcos, California, USA) designated as groups A, B, and C. The distal end cuts of the wires were preserved before subjecting them to the oral environment for canine retraction. Nance palatal arch and Gurin locks (Dental Morelli Ltd., Jardim Saira, Brazil) were used for augmenting anchorage. The inserted archwires were recovered and decontaminated with 70% isopropyl alcohol [8] (Coral Clinical systems, Verna, Goa, India) to remove any microbial contaminants before testing, and the same was performed for control samples.

### 2.4. Sample Testing

The tension test was conducted on the ten samples of 30 mm length using an Instron Universal Testing Machine (Model 3366, Instron Corp., High Wycombe, UK), having 15 kN load cell. A pair of grips with grooves was used for holding the wire specimens at approximately 10 mm gauge length, and the tension test was performed at a crosshead speed of 1 mm per minute [6].

**Figure 1:**
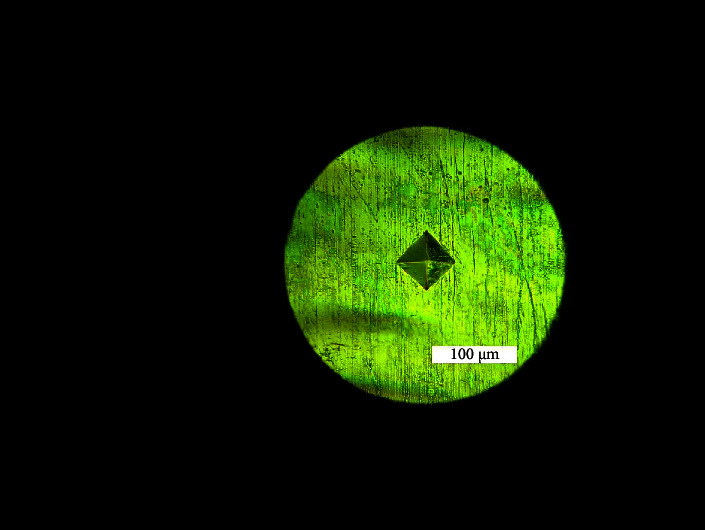
Indent formed with Vickers microhardness indenter.

Vickers microhardness measurements were performed at 25°C with a 9.81 Newton load and 15 seconds dwell time [9] for all specimens with a Matsuzawa hardness tester (MMT-X7A, Matsuzawa Co., Ltd, Japan). The average of three readings was considered as the microhardness value for each sample. The indent image is illustrated in Figure 1. For comparison with the hardness values of nanoindentation, the Vickers hardness values were converted to GPa [6].

**Figure 2:**
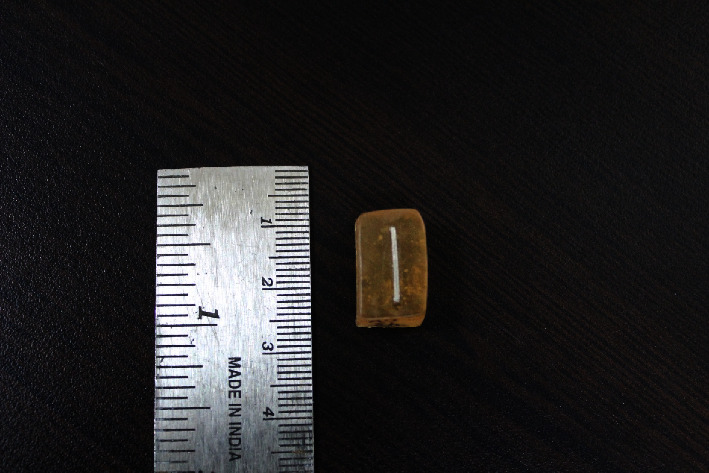
Samples embedded in polymethyl methacrylate resin.

**Figure 3:**
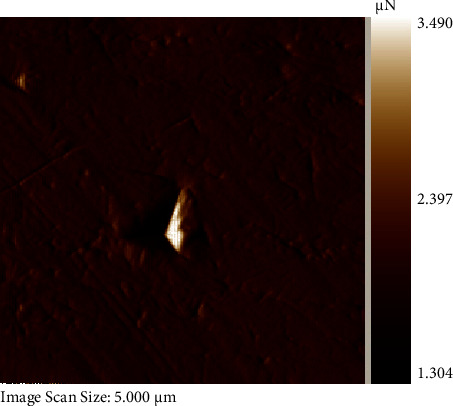
Indents formed by nanoindenter.

For nanoindentation testing, the wires were cut (10 mm length) with a low-speed water-cooled diamond disc to prevent work hardening and embedded in polymethyl methacrylate resin [6] (Figure 2) and polished with colloidal silica (particle size 0.04 microns) to achieve surface roughness of less than about 200 nm to obtain significant results. The specimen was set on a resin stage with cyanoacrylate glue (Fevikwik, Pidilite Industries, Mumbai, Maharashtra, India). Nanoindentation testing was operated at 25–32°C with the Hysitron® TI 750-D Ubi-1. Each test consisted of 3 segments: 10 seconds for loading to the peak value, 1 second holding at the peak load, and 10 seconds for unloading. A peak load of 100 mN was used for the measurements [6]. The nanoindentation indent image is illustrated in Figure 3. Hardness and elastic modulus were deciphered from the software supported by the nanoindenter.

The tension test and Vickers microhardness tests were performed at baseline and 15 weeks (10 samples/time point/group). The nanoindentation test was done on two samples in each group and the means were compared with VHN values.

### 2.5. Statistical Analyses

All statistical analyses were performed using the Statistical Package for Social Sciences (SPSS released in 2009, PASW Statistics for Windows, version 18.0, SPSS Inc., Chicago). Normality was tested using the Shapiro–Wilk test. ANOVA with the post hoc Games Howell test was used for intergroup comparisons at baseline and 15 weeks for MOE, YS, SF, and VHN. The paired *t*-test was used for intragroup comparisons between baseline and 15 weeks for MOE, YS, SF, and VHN. The mean percentage reduction in the values of MOE, YS, SF, and VHN was calculated ((baseline−15 weeks)/baseline *∗* 100) and was compared among the groups using Kruskal–Wallis ANOVA with the post hoc Dunn test. A *P* value of <0.05 was considered statistically significant.

## 3. Results

There were no significant differences in the mean modulus of elasticity, yield strength, and spring factor among the three groups at baseline and 15 weeks. Intragroup comparisons showed a significant reduction in the mean MOE at 15 weeks compared with baseline in all three groups. A significant reduction in yield strength was seen in group A at 15 weeks compared to baseline (*P*=0.025), while no significant difference was seen in groups B and C (*P*=0.136 and 0.093). There were no significant differences between baseline and 15-week spring factor values in all the groups (*P*=0.864, 0.505, and 0.591) (Table 2).

There was a significant marginal difference in the mean VHN in the three groups at baseline (*P*=0.048). However, the post hoc test showed no significant differences. Similarly, at 15 weeks, there was a significant difference in the mean VHN in the groups (*P* < 0.001). Post hoc tests revealed that group B had a significantly higher mean than groups A and C. A significant reduction in VHN was seen in groups B and C at 15 weeks compared to baseline (*P*=0.001 and 0.031), while no significant difference was seen in group A (*P*=0.666).

The mean percentage reduction in the values of MOE, YS, SF, and VHN was calculated ((baseline−15 weeks)/baseline *∗* 100) and was compared among the groups (Table 3). There was no significant difference in the mean percentage reduction of MOE, YS, and SF among the three groups (*P*=0.144, 0.093, and 0.324), respectively. However, there was a significant difference in the mean percentage reduction in VHN among the three groups. The post hoc test showed that the percentage reduction was higher in group B than in groups A and C.

Nanoindentation (Table 4) illustrated that Young's modulus decreased over 15 weeks in groups A and C, but B observed an increase. Hardness decreased in all groups over 15 weeks (Table 4). A wide variation exists in the hardness and Young's modulus values obtained by nanoindentation when compared to those obtained from the tension test and the Vickers hardness test.

**Figure 4:**
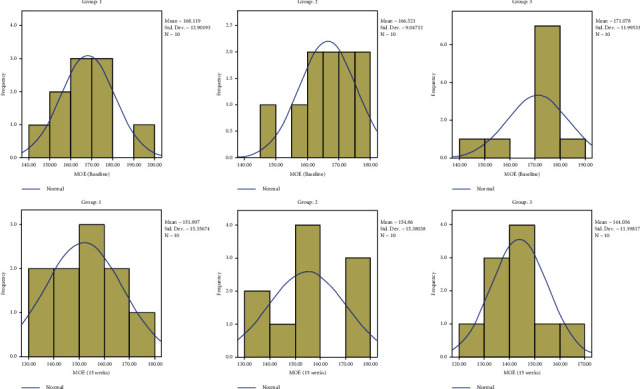
Comparison of MOE.

**Figure 5:**
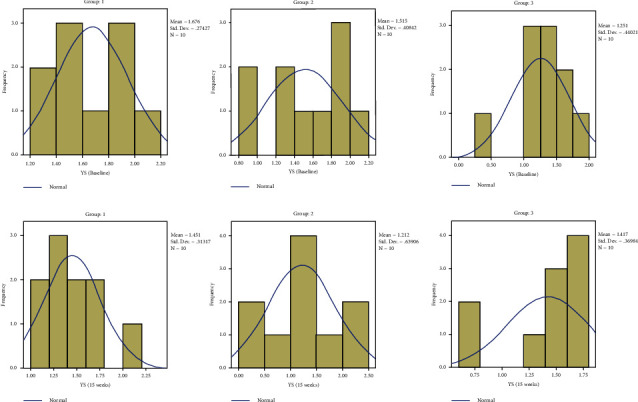
Comparison of yield strength.

**Figure 6:**
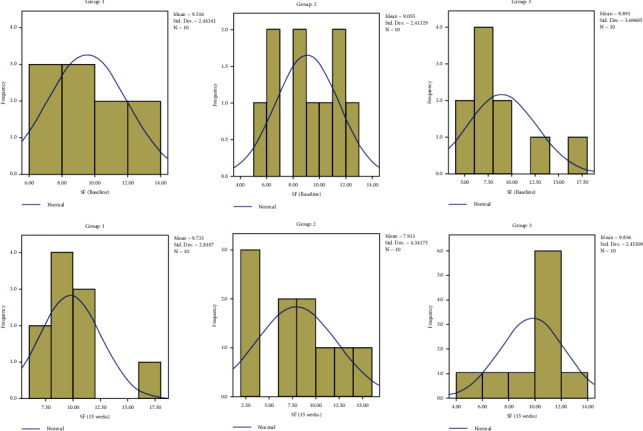
Comparison of SF.

**Figure 7:**
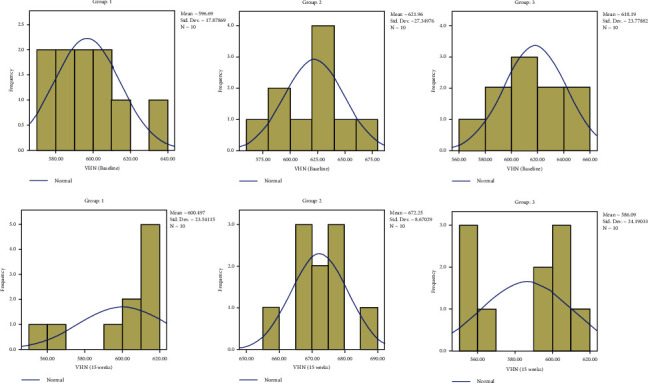
Comparison of VHN.

The results are depicted in a series of figures with histograms (Figures 4–7).

## 4. Discussion

This study was conducted to evaluate changes in the mechanical properties of 3 commercially manufactured preformed stainless steel wires, after 15 weeks of intraoral exposure.

The wire was not altered in any way as elastic modulus is affected by the amount of cold working. Thereby, Gurin locks and Nance palatal arches were used to augment anchorage.

Young's modulus denotes the rigidity of the material. Hence, the higher the value, the stiffer the wire will be [10]. At baseline, the values for Young's modulus of elasticity ranged from a mean of 168.12 to 171.08 GPa. This was greater than the value obtained in the past literature [2, 7]. The values obtained in this study corroborate with the review performed by Kapila and Sachdeva [3], who have observed values ranging from 168.3 to 172.4 GPa in various studies. The modulus of elasticity decreased during 15 weeks of the study and the change was statistically significant in all groups. Hence, rigidity of the wires decreases with continued use.

Yield stress represents the stress value at which 0.1% or 0.2% of plastic strain has occurred and is important in the evaluation of stress at which permanent deformation of the wire begins. At baseline, the yield stress at 0.2% offset ranged from a mean of 1.25 to 1.68 GPa. These values are supported by prior studies [2, 3] but differ from a study [11] that obtained a mean of 2.8 GPa. The values of yield stress decreased over 15 weeks in groups A and B but increased in C. However, only the change in A was statistically significant.

Spring factor indicates the clinical performance of wires from the perspective of working range [12]. At baseline, the values for the spring factor ranged from a mean of 8.89 to 9.52. Drake et al. [2] obtained a mean of 9.3 for the 3M Unitek wires. Findings of this study comply with a review by Kapila and Sachdeva [3], who noted a range of 7.7 to 11 across various studies. The values of spring factor increased during 15 weeks in groups A and C, but the change was not statistically significant in any group.

Kusy et al. [13] have implied that as wire ages, its hardness decreases, and hence, the coefficient of friction offered by the archwire increases. At baseline, the Vickers hardness test achieved a mean hardness value ranging from 600.5 to 672.25 kgf/mm^2^ (5.88 to 6.59 GPa). Oh et al. [14] found that for stainless steel archwires, the hardness values varied in the range from 456 to 586 kgf/mm^2^, whereas in this study, the values were higher. Hardness of A. J. Wilcock stainless steel wire has been shown to display a mean of 602 kgf/mm^2^ [9], which falls in the range of this study. The decrease in VHN was statistically significant in groups B and C.

The reasons for change in mechanical properties of orthodontic archwires can be many. After intraoral aging in NiTi, degraded performance, limited elasticity, and decreased ultimate tensile strength have been observed [15]. This is caused by altered structural and compositional characteristics.

Wires when immersed in fluoride solutions exhibited loss of surface material and fluoride-related disruption of the protective oxide layer [16]. This can cause hydrogen absorption, stress corrosion cracking, and embrittlement. Wires remained in the oral cavity for 15 weeks. During this phase, they would, invariably, have been exposed to topical fluoride, fluoridated water, and toothpastes. This could also play a role in the differences in the mechanical properties observed. Trace elements in an alloy, existence of different phases of metal, and diverse surface conditions can play a role in the variation in mechanical properties [17].

Nanoindentation tests revealed the mean values of modulus of elasticity at baseline, to be between 117.6 and 128.3 GPa. This was lower than the values obtained in the previous studies [6, 8]. The mean values of hardness achieved by nanoindentation were 4.5 to 7.2 GPa, which supports previous studies [6, 8]. The wide difference in the values obtained could be due to different manufacturing processes followed by different companies. The differences in temperature and loads used during testing may also play a role.

The changes in the properties obtained from nanoindentation may not be representative of identical changes all through the bulk of the archwires. The changes observed at the surface and in the bulk may contradict each other. The changes in elastic modulus, hardness, and surface topography may have a considerable effect on orthodontic tooth movement, despite being localized to the surface. This is because movement is determined largely by the surface, together with the bulk interactions between the bracket system and archwires [8].

The Vickers hardness values were converted to compare with the nanoindentation test values. At baseline, the values were seen to range from 5.88 to 6.59 GPa which was different from an earlier study [6]. A passive chromium oxide (Cr_2_O_3_) protective layer which is formed on the surface of stainless steel wires may be responsible for these differences [5]. Fracture of the oxide film may control the yield under the nanoindenter before the initiation of plastic deformation [6]. Hence, the oxide film thickness affects the mechanical properties. Nanoindentation assesses a much smaller volume of material, and this may explain the differences in mechanical properties obtained. The stress distribution at the nanoindenter tip is complex compared to the much simpler stress distributions in the macroscopic tension tests. There could also be significant differences between the near-surface mechanical properties and bulk mechanical properties [6].

Lower values of modulus of elasticity provide the ability to apply lower and more constant forces over time as the appliance experiences deactivation [3]. However, a decrease in the modulus of elasticity decreases wire stiffness, thereby decreasing torque [5]. In the finishing stages, appropriate stiffness in archwire at relatively small deflection rather than range of activation is the primary consideration. A steel archwire is invariably needed for full torque expression.

Any mechanical deformation of the wire transforms the austenitic phase with higher elastic modulus to a martensitic phase with lower values of elastic modulus [18, 19]. Further research is needed to investigate the difference in mechanical properties obtained at the surface and within the bulk of orthodontic wires in nanoindentation testing and the association with microstructural variations. Wire alternatives with beta-titanium archwires and even multistranded archwires may be used to compare the mechanical properties of stainless steel archwires.

A limitation of the study was the smaller sample size due to restricted resources available. Clinical significance of the consequences of this study needs additional research for further evaluation and clarification. Torsion testing could have been performed to study the change in mechanical properties. In future, researchers may study flexural strength and fretting wear.

## 5. Conclusion

Young's modulus and hardness decreased for stainless steel wires from all manufacturers. The changes in the spring factor were not of statistical significance. The values of Young's modulus and hardness obtained from the tension test, Vickers hardness test, and nanoindentation test did not produce identical results. However, the hardness values obtained from both tests showed a decrease after 15 weeks. This could be due to the fact that nanoindentation test analyses a very small sample where surface and bulk properties of wires may produce microstructural variations.

## Figures and Tables

**Table 1:** Abbreviations.

Abbreviation	Description
MOE	Modulus of elasticity
YS	Yield strength
SF	Spring factor
VHN	Vickers hardness number

**Table 2:** Comparison of modulus of elasticity, yield strength, spring factor, and VHN.

	Group A	Group B	Group C	*P* value
	Mean ± SD	Mean ± SD	Mean ± SD	
MOE (GPa)				
Baseline	168.12 ± 12.90	166.52 ± 9.05	171.08 ± 12	0.668; NS
15 weeks	151.9 ± 15.36	154.86 ± 15.38	144.06 ± 11.20	0.228; NS
*P* value	0.025; Sig	0.054; Sig	0.004; Sig	
YS (GPa)				
Baseline	1.68 ± 0.27	1.52 ± 0.41	1.25 ± 0.44	0.058; NS
15 weeks	1.45 ± 0.31	1.21 ± 0.64	1.42 ± 0.37	0.469; NS
*P* value	0.025; Sig	0.136; NS	0.093; NS	
SF				
Baseline	9.52 ± 2.44	9.06 ± 2.41	8.89 ± 3.70	0.884; NS
15 weeks	9.73 ± 2.82	7.91 ± 4.34	9.84 ± 2.45	0.357; NS
*P* value	0.864; NS	0.505; NS	0.591; NS	
VHN (GPa)				
Baseline	600.50 ± 23.54	672.25 ± 8.67	618.2 ± 23.80	0.05; NS
15 weeks	596.70 ± 17.90	622.00 ± 27.30	586.09 ± 24.19	<0.001; Sig
*P* value	0.666; NS	0.001; Sig	0.031; Sig	

NS, not significant; Sig, significant.

**Table 3:** Intergroup comparison of the mean percentage reduction in the values of MOE, YS, SF, and VHN was calculated ((baseline−15 weeks)/baseline *∗* 100).

	Group A	Group B	Group C	*P* value
	Mean ± SD	Mean ± SD	Mean ± SD	
MOE	9.26 ± 10.25	6.82 ± 9.95	15.03 ± 12.74	0.144; NS
YS	11.08 ± 25.63	11.78 ± 52.43	−28.63 ± 59.04	0.093; NS
SF	−9.04 ± 44.77	5.15 ± 56.55	−25.66 ± 45.83	0.324; NS
VHN	−0.51 ± 4.57	−7.46 ± 4.41	5.70 ± 7.05	<0.001; Sig

NS, not significant; Sig, significant.

**Table 4:** Descriptive statistics with nanoindentation: modulus of elasticity and hardness.

	Group A	Group B	Group C
	Mean	Mean	Mean
MOE (GPa)			
Baseline	124.45	117.60	128.30
15 weeks	119.66	118.20	107.17
Hardness (GPa)			
Baseline	6.25	4.50	7.20
15 weeks	5.35	4.10	6.16

## Data Availability

Data used to support this study are available on request from the corresponding author.
